# Exploring the Consistency of the Quality Scores with Machine Learning for Next-Generation Sequencing Experiments

**DOI:** 10.1155/2020/8531502

**Published:** 2020-02-25

**Authors:** Erdal Cosgun, Min Oh

**Affiliations:** ^1^Microsoft Genomics Team, Redmond, WA, USA 98052; ^2^Virginia Tech University, Dep. of Computer Science, Blacksburg, VA 24061, USA

## Abstract

**Background:**

Next-generation sequencing enables massively parallel processing, allowing lower cost than the other sequencing technologies. In the subsequent analysis with the NGS data, one of the major concerns is the reliability of variant calls. Although researchers can utilize raw quality scores of variant calling, they are forced to start the further analysis without any preevaluation of the quality scores.

**Method:**

We presented a machine learning approach for estimating quality scores of variant calls derived from BWA+GATK. We analyzed correlations between the quality score and these annotations, specifying informative annotations which were used as features to predict variant quality scores. To test the predictive models, we simulated 24 paired-end Illumina sequencing reads with 30x coverage base. Also, twenty-four human genome sequencing reads resulting from Illumina paired-end sequencing with at least 30x coverage were secured from the Sequence Read Archive.

**Results:**

Using BWA+GATK, VCFs were derived from simulated and real sequencing reads. We observed that the prediction models learned by RFR outperformed other algorithms in both simulated and real data. The quality scores of variant calls were highly predictable from informative features of GATK Annotation Modules in the simulated human genome VCF data (R2: 96.7%, 94.4%, and 89.8% for RFR, MLR, and NNR, respectively). The robustness of the proposed data-driven models was consistently maintained in the real human genome VCF data (R2: 97.8% and 96.5% for RFR and MLR, respectively).

## 1. Introduction

**Figure 1:**
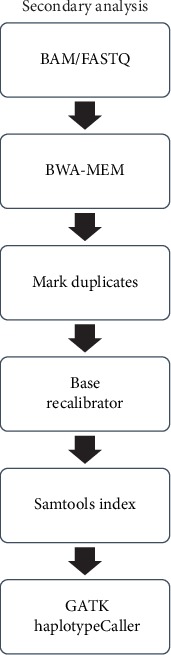
Analysis pipeline of secondary analysis of NGS data.

With the development of next-generation sequencing (NGS) technology, progress has been made in the field of bioinformatics. The most important reason for this is that this new data type is large and classical methods are slow and not repeatable for doing the relevant analysis. Analysis pipeline of NGS data is given in Figure 1. The most important stage of this process is the stage of the alignment and variant calling defined as secondary analysis. Industry standard of the secondary analysis pipeline is Burrows-Wheeler Aligner (BWA) and the Genome Analysis Toolkit (GATK), which are part of the Broad Institute's best practices analysis pipeline [1–4]. The GATK best practices provide step-by-step recommendations for performing variant discovery analysis in high-throughput sequencing (HTS) data [1]. One of the most important data obtained from this pipeline is Variant Calling Format (VCF) file.

VCF is a text file format (most likely stored in a compressed manner). It contains metainformation lines, a header line, and then data lines each containing information about a position in the genome and for storing gene sequence variations. The format also has the ability to contain genotype information on samples for each position [5]. VCF files are flexible with eight fixed fields including chromosome (CHROM), position (POS), known variant IDs such as dbSNP identifications (ID), reference allele (REF), alternate allele(s) (ALT), variant quality score (QUAL), filter information summarizing why a variant was or was not considered valid by the variant calling software (FILTER), and an information field (INFO) [6]. One of the major parameters of VCF files is Phred-scaled quality score (QUAL). 1000 Genomes Project defined VCF as follows.

QUAL Phred-scaled quality score for the assertion made in ALT, i.e., give -10log_10 prob (call in ALT is wrong). If ALT is “.” (no variant), then this is -10log_10 p(variant), and if ALT is not “.,” this is -10log_10 p (no variant). High QUAL scores indicate high confidence calls. Although traditionally people use integer Phred scores, this field is permitted to be a floating point to enable higher resolution for low-confidence calls if desired [7]. The importance of QUAL data came from its meaning: QUAL tells us how confident we are that there is some kind of variation at a given site. The variation may be present in one or more samples [7, 8].

Quality scores can be analyzed and their distributions can be checked with different tools such as SAMtools [9] and R-Bioconductor VariantAnnotation package [10]. BWA+GATK best practices will create VCF files with QUAL, and researchers are using this data for filtering, merging the variations in their studies. Therefore, QUAL is one of the important parameters of VCF files.

Machine learning prediction algorithms are very helpful for understanding and extracting the relevant information of genomics data sets. Wood et al. developed a machine learning approach called Cerebro that increased the accuracy of calling validated somatic mutations in tumor samples from cancer patients [11]. Trakadis et al. present a novel predictor which could potentially enable studies exploring disease-modifying intervention in the early stages of the disease with supervised machine learning (ML) methods [12]. Kawash et al. present ARIADNA (ARtificial Intelligence for Ancient DNA), a novel approach based on machine learning techniques, using specific aDNA characteristics as features to yield improved mutation call [13]. We can give different examples of using ML on genomics.

In this study, we developed a ML model that can predict the “expected QUAL scores of VCF files.” The reason behind this aim is researchers directly using the results of secondary analysis pipelines without checking the parameters. On the other hand, repeatability of the secondary analysis is a big question for most of the implementations. If researchers do not have “concordant secondary analysis pipelines,” they will have different VCF files. This will directly affect the QUAL scores which are used for filtering and QC purposes. Researchers can predict the expected QUAL scores and our model and trust their VCF files.

## 2. Materials and Methods

### 2.1. Data Set

In order to assess the predictive power of the regression models, synthetic human genomes, as well as real human genomes, were used in our evaluation. The simulation of the human genomes was conducted with the sophisticated genomic simulator, VarSim, considering the full spectrum of variants [14]. The VarSim perturbs human reference genome by injecting a wide range of variants, including single-nucleotide variants, small indels, and large structural variants into the reference genome. In total, twenty-four diploid genomes were synthesized based on GRCh37/hg19 human genome assembly with germline mutations which are sampled from comprehensive genetic variation databases (dbSNP and DGV) [15, 16]. The execution parameters adjusting the number of each variant type of synthetic genome were varied according to the distributions of the number of variants of five different continental groups reported by the 1000 Genomes Project [17]. Sequentially, we simulated next-generation sequencing reads based on the synthetic human genomes using ART, which was used as a primary tool for the simulation study of the 1000 Genomes Project [18]. The empirical read quality profile derived from large real sequencing data was utilized to simulate sequencing errors when ART simulated paired-end Illumina sequencing reads with ×30 coverage.

In addition to synthetic human genomes, real human genome sequencing reads were secured from the Sequence Read Archive (SRA) of the National Center for Biotechnology Information (NCBI) [19]. We collected the twenty-four whole-genome sequencing reads which resulted from Illumina paired-end sequencing with at least ×30 coverage using SRA toolkit 2.8.2.

### 2.2. Variant Calling

For both simulated and real sequencing reads, variants were called using Microsoft Genomics Service which is an advanced optimization of Broad Institute's best practices pipeline for genome reassembly—the Burrows-Wheeler Aligner (BWA) and the Genome Analysis Toolkit (GATK). Microsoft Genomics Service accelerates the secondary analysis of genomes by fully parallelizing alignment and variant calling process on the cloud hardware. The sequencing reads were aligned to the human reference genome (GRCh37/hg19), and the genotypes were called with parallelization of GATK HaplotypeCaller supported by Microsoft Genomics Service.

### 2.3. Transforming Variant Calling Format into Machine Learnable Structure

The variant calls of the secondary analysis powered by Microsoft Genomics Service were obtained in the form of variant call format (VCF). We have used R-Bioconductor “VariantAnnotation” [10] package to import VCF files into an R environment and to convert the imported VCF data into a data frame object in which INFO and genotype fields and their values for each variant were extracted as well as FIXED fields.  Table 1 shows the details of various fields that were used as learning features of each called variant for the following machine learning step.

### 2.4. Building Predictive Models

**Figure 2:**
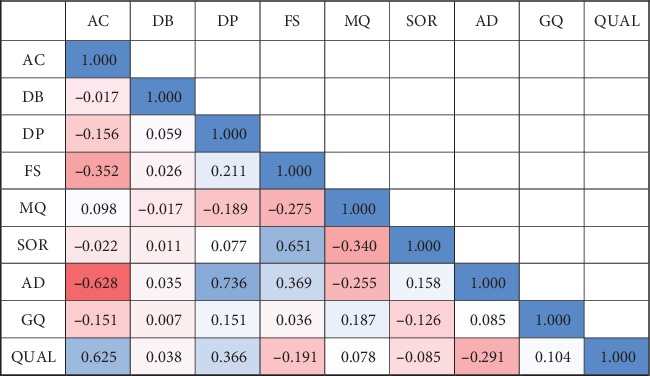
Average correlation coefficient matrix of simulated 24 data sets.

**Figure 3:**
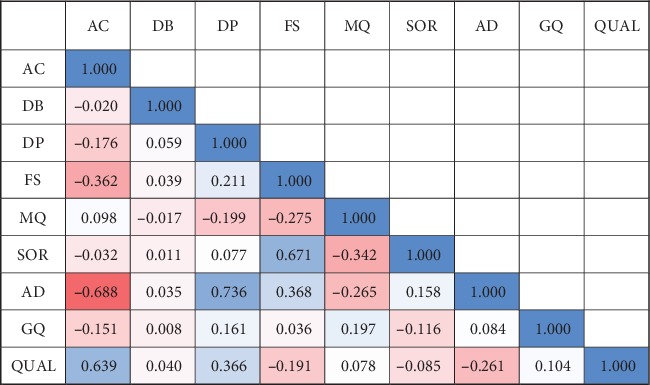
Average correlation coefficient matrix of real 24 data sets.

Prediction models were trained on VCF data containing various fields derived from the variant caller. These fields accommodating the technical values of GATK Annotation Modules for each variant call were used as features for regression models to estimate quality score. We empirically selected informative features, including “Allele count” (AC), approximate read depth (DP), and allelic depths (AD) based on correlations between quality score and other features averaged across the simulated samples (Figure 2). To avoid unfair prediction, it was prohibited to include Phred-scaled likelihoods (PL) in learning features as it directly derives quality score. Also, duplicated features that are simply statistics of other features or scaled values originated from other features were excluded in feature selection step. Adjusted Pearson correlation coefficients for selected features are as follows: simulated data set: AC (0.625), DP (0.366), and AD (-0.291) (Figure 2); real data set: AC (0.639), DP (0.366), and AD (-0.261) (Figure 3).

We applied three machine learning algorithms to estimate a quality score of each variant call: multivariate linear regression, random forest regression, and neural network regression. Multivariate linear regression is simple and widely used as a basic step in regression analysis, which attempts to model a linear relationship between multiple independent variables and dependent variable by minimizing estimation errors. Random forest regression is a nonparametric regression method that aggregates results of regression trees. Random forest regression algorithm draws bootstrap samples from the original data and grows regression tree for each of the bootstrap samples by choosing the best split at each node from randomly sampled predictors. We obtained optimal parameters of the number of trees in a forest (ntree) and the number of randomly sampled predictors that are used as candidates for best split at each node (mtry) using partially manual grid search (ntree = 10 and mtry = 2). Neural network regression was conducted by constructing a neural network which consists of input, hidden, and output layers, sequentially. Each layer contains one or multiple nodes, and each of which receives a weighted summation of its inputs in the preceding layer.

The weights of nodes are randomly assigned in an initial stage and are adjusted in each iteration until the output layer produces an optimal solution or the iteration reaches the maximum iteration boundary. Each node in the input layer takes the value of each variable in the input data vector. The output layer has a single output node estimating a quality score. With partially manual grid search, we obtained optimal parameters for learning task: node size of the learning rate = 0.01, hidden layer = 12, maximum iteration = 500, and decay = 0.01.

In a learning scheme, each VCF data is split into training data containing 80% of whole data points and test data having the rest of the data points. Regression models were trained on the training data and evaluated on the test data. Prediction performance of regression models was evaluated with five measurements, including R squared (*R*^2^), root-mean-square error (RMSE), relative squared error (RSE), mean absolute error (MAE), and relative absolute error (RAE).

## 3. Results

We built data-driven predictive models for estimating quality scores of variant calls in VCF data derived from 24 simulated human genome reads and 24 real human genome reads using supervised machine learning techniques. Three learning algorithms, multivariate linear regression (MLR), random forest regression (RFR), and neural network regression (NNR), were used to train models based on the most informative features of GATK Annotation Modules for prediction of quality scores. *R*^2^ and error measurements were averaged over the simulated data set as shown in Table 2. The result shows that quality scores of variant calls are highly predictable from informative features of GATK Annotation Modules in simulated human genome VCF data. Interestingly, the prediction models learned by RFR outperform other models with an average *R*^2^ of 96.7%. The other algorithms, MLR and NNR, also represent high predictive power (94.4% for MLR and 89.8% for NNR). In addition, RFR achieved lowest error in all four error measurements, including RMSE, RSE, MAE, and RAE.

Robustness of the proposed data-driven models for predicting quality scores was consistently maintained in VCF data of real human genomes (Table 3). The highest predictive accuracy was achieved by models derived from RFR (average R2 of 97.8%). In addition, MLR models show a relatively high accuracy (an average *R*^2^ of 96.5%), whereas NNR models present a relatively low accuracy (an average *R*^2^ of 59.3%). RFR and MLR models trained on real VCF data resulted in better performance than those models trained on simulated VCF data, although the averaged R2 of NNR models trained on real VCF data was decreased. Accuracies of NNR models had largely fluctuated across the real VCFs (standard deviation of *R*^2^: 34.5%) as well as simulated ones (standard deviation of *R*^2^: 19.1%). Furthermore, two error measurements, RMSE and RAE, of the models trained on real VCF data had been increased when compared to that of the other models trained on simulated data, while both MLR and RFR models of real VCF data show the reduced RSE and MAE than that of the corresponding models of simulated VCF data.

## 4. Discussion

In this study, we have predicted the expected QUAL scores with three different ML methods. Based on our results, RF showed the highest accuracy for the predictions on test sets. Researchers can develop their own models with their retrospective VCF databases. Once they have their VCF files from the secondary analysis pipelines, they can use AC, DP, and AD for checking the performance of the pipelines. In general, for the benchmark, quality scores can be useful for performance assessment. Although we have shown our ML results, we recommend that users can predict their own QUAL values with publicly available data like NA12878 (http://www.internationalgenome.org/data-portal/sample/NA12878).

Another output of the study is showing the difference of simulated and real data on secondary analysis. There are different simulation approaches in the literature [14–18] but there is no consensus for the effect of the simulation algorithms on alignment and variant calling phase. In our study, we have showed that neural network's performance was dramatically decreased on real data set. These results will be helpful for the researchers who are going to do simulation and use NN for prediction. On the other hand, MLR is a baseline model for most of the regression studies. In our study, we showed that MLR is still valid and useful for predicting the QUAL on NGS data sets. MLR has statistical assumptions but we have used three parameters and they satisfied the assumptions.

Another importance of the approach we used in our study is the possibility of generalization for other VCF parameters (DP and QD). In this way, it is possible to perform supportive analyses especially for VCF concordance analysis. When the secondary analysis studies in the literature are examined, the effects of the parameters on the ML models are ignored. In our study, a new perspective has been introduced to the researchers who are planning to work on this subject. Whole-genome data sets were used in this study. We are also working on developing ML models for exome and other panel genome data with the same approach.

## 5. Conclusion

In summary, we introduced a secure and scalable quality control workflow for sequence alignment and cohort level germline variant calling for SNPs and indels. Our approach leverages the elastic computing and largely follows common bioinformatics best practices for germline variant calling. Our optimized implementations of specific components accelerate the research projects, shortening the concordance analysis time from sequence data to cohort level variant calls.

The most important problem of genetic studies in recent years is repeatability, consistency, and high cost. Thanks to this study, genetic data control can be performed easily in research projects with budget constraints. The proposed approach is to make experiments that can be repeated more difficult, rather than predicting an already known variable: quality score. In particular, such cloud-based algorithms are expected to produce much cheaper and much more reliable tools with machine learning and deep learning.

## Figures and Tables

**Table 1:** VCF fields extracted by VariantAnnotation package.

Category	Field	Type	Description
INFO	AC	Integer	Allele count in genotypes, for each ALT allele, in the same order as listed
	DB	Flag	dbSNP membership
	DP	Integer	Approximate read depth: some reads may have been filtered
	FS	Float	Phred-scaled *p* value using Fisher's exact test to detect strand bias
	MQ	Float	RMS mapping quality
	SOR	Float	Symmetric odds ratio of 2 × 2 contingency table to detect strand bias

GENO	AD	Integer	Allelic depths for the ref and alt alleles in the order listed
	GQ	Integer	Genotype quality

FIXED	QUAL	Float	A quality score associated with the inference of the given alleles

**Table 2:** Prediction result for 24 simulated VCFs.

Metric	Machine learning algorithm	*R* ^2^	RMSE	RSE	MAE	RAE
Average	MLR	0.944	181.726	0.056	114.624	0.196
	**RFR**	**0.967**	**139.537**	**0.033**	**49.050**	**0.084**
	NNR	0.898	212.891	0.102	110.602	0.189

Standard deviation	MLR	0.003	4.346	0.003	3.180	0.007
	RFR	0.004	8.387	0.004	1.254	0.003
	NNR	0.191	124.380	0.191	100.858	0.172

MLR: multivariate linear regression; RFR: random forest regression; NNR: neural network regression; RMSE: root-mean-square error; RSE: relative squared error; MAE: mean absolute error; RAE: relative absolute error.

**Table 3:** Prediction result for 24 real VCFs.

Metric	Machine learning algorithm	*R* ^2^	RMSE	RSE	MAE	RAE
Average	MLR	0.965	250.403	0.036	88.170	0.302
	**RFR**	**0.978**	**205.793**	**0.023**	**26.474**	**0.085**
	NNR	0.593	804.970	0.409	180.281	0.599

Standard deviation	MLR	0.015	44.463	0.015	12.665	0.081
	RFR	0.005	39.842	0.005	7.604	0.013
	NNR	0.345	448.786	0.344	91.056	0.286

## Data Availability

Real and Simulated data can be found from: https://aka.ms/genomicseducation.
